# Toxic effects of GAL80 and RNAi against fluorescent proteins on motor performance in *Drosophila*

**DOI:** 10.17912/micropub.biology.001943

**Published:** 2025-12-05

**Authors:** Kathryn Mizzi, Francesca Grech, Sylvana Tabone, Rebecca Cacciottolo, Ruben J Cauchi

**Affiliations:** 1 Department of Physiology and Biochemistry, University of Malta, Msida, L-Imsida, Malta; 2 Centre for Molecular Medicine and Biobanking, University of Malta, Msida, L-Imsida, Malta

## Abstract

Transgenic regulators and reporter controls such as GAL80 and RNA interference (RNAi) constructs targeting fluorescent proteins are invaluable tools in *Drosophila* research. During experiments designed to temporally regulate gene expression, we observed unexpected flight defects in aged flies with constitutive expression of temperature-sensitive GAL80 ^TS^ . Furthermore, ubiquitous expression of RNAi targeting EGFP or mCherry induced survival and motor impairments, even in the absence of corresponding fluorescent reporters. These findings suggest that genetic tools commonly assumed to be physiologically inert can have measurable effects on motor behaviour. Researchers employing these tools should therefore interpret behavioural data with caution and include appropriate controls.

**Figure 1. Effect of GAL80 or RNAi constructs targeting fluorescent reporters EGFP and mCherry on motor behaviour in flies:**
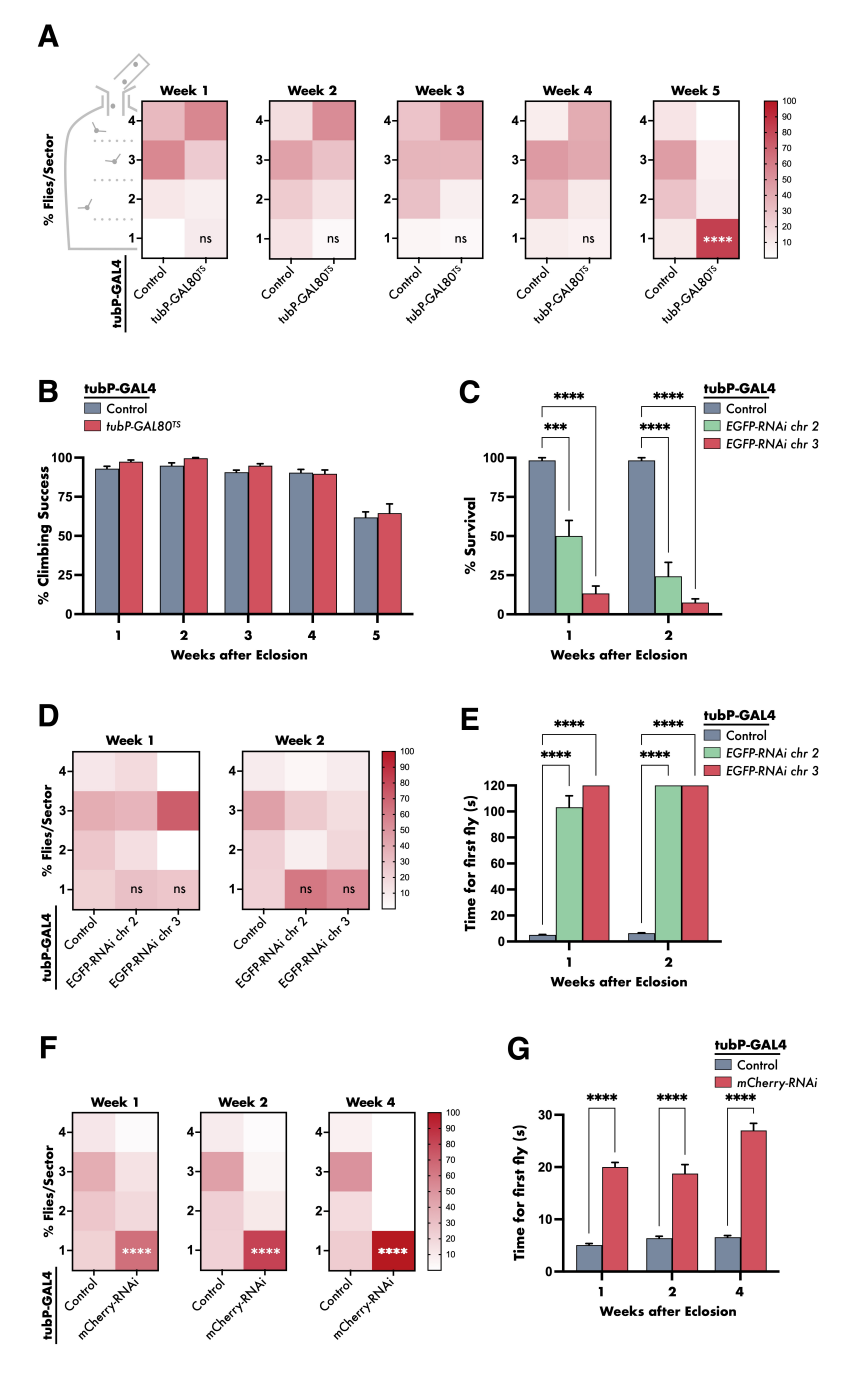
( **A** ) Heat maps comparing distribution of final landing position (Sector 4, top; Sector 1, bottom) of male flies with ubiquitous expression of GAL80 ^TS^ compared to driver-only control, incubated at 29 ^o^ C ( *n* = 60-300/genotype). Constitutive GAL80 ^TS^ expression induced flightlessness (increased distribution of flies at Sector 1) on week 5 post-eclosion. ( **B** ) Bar chart showing that the climbing ability of flies with ubiquitous GAL80 ^TS^ expression, assessed at different timepoints, was comparable to that of the driver-only control ( *n* = 60-300/genotype). ( **C** ) Bar chart showing percentage number of flies still alive on week 1 and week 2 post-eclosion. Flies with constitutive expression of RNAi constructs targeting EGFP exhibited decreased survival compared to the driver-only control ( *n* = 60-90/genotype). ( **D** ) Heat maps showing that distribution of final landing position of week 1- and week 2-old male flies with ubiquitous expression of EGFP-targeted RNAi was not significantly different from that of the driver-only control ( *n* = 60-90/genotype). ( **E** ) Bar chart showing the time taken for the first fly in a sampled population to climb to a pre-determined threshold on week 1 and week 2 post-eclosion. Flies expressing RNAi targeting EGFP ubiquitously took significantly longer compared to the driver-only control ( *n* = 45-60/genotype). ( **F** ) Heat maps comparing distribution of final landing position of male flies with ubiquitous expression of mCherry-targeted RNAi compared to driver-only control, as determined on multiple timepoints post-eclosion ( *n* = 60/genotype). Expression of RNAi targeting mCherry induced a significant number of non-fliers on all timepoints. ( **G** ) Bar chart showing the time taken for the first fly in a sampled population to climb to a pre-determined threshold, assessed at multiple timepoints following eclosion. Flies expressing RNAi targeting mCherry exhibited prolonged times compared to the driver-only control ( *n* = 60/genotype). For graphs, each bar represents the mean ± SEM of several independent experiments. Statistical significance was tested using a two-way ANOVA with Bonferroni’s *post hoc* test. For all data, ns = not significant, *** *p* < 0.001, and **** *p* < 0.0001.

## Description

The yeast-derived GAL4/UAS system has transformed *Drosophila* genetics by enabling targeted spatial control of transgene expression (Brand & Perrimon, 1993). The integration of GAL80, a transcriptional repressor that interacts with the GAL4 activation domain, further enhanced this tool by adding a layer of temporal control, thereby allowing dynamic regulation of gene expression (Cauchi & van den Heuvel, 2006; Duffy, 2002; Lee & Luo, 1999). For experiments requiring broad or constitutive transgene expression, one of the most frequently utilised GAL4 drivers is the *α-tubulin* -GAL4 ( *tubP* -GAL4) driver, which expresses GAL4 under the control of the *αTub84B* gene promoter (O'Donnell et al., 1994). The same promoter is also widely used to drive GAL80 ubiquitously during development and across tissues, thus facilitating temporal control on constitutive or conditional GAL4 expression (Lee & Luo, 1999). RNA interference (RNAi) constructs targeting fluorescent proteins such as Enhanced Green Fluorescent Protein (EGFP) and mCherry, which are absent from the *Drosophila* proteome, are also routinely employed as experimental controls (Neumuller et al., 2012). By engaging the RNAi machinery without targeting endogenous transcripts, these constructs are presumed to have no physiological impact. However, during years of experiments designed to spatially and temporally regulate gene expression in *Drosophila* , we observed unexpected behavioural defects in flies carrying GAL80 or RNAi constructs targeting fluorescent reporters EGFP and mCherry.

We and others commonly employ the temperature-sensitive GAL80 (GAL80 ^TS^ ) tool to achieve temporal suppression of transgene expression during development, with reactivation restricted to adult flies following eclosion (McGuire et al., 2004). In this approach, flies are typically maintained at permissive temperatures (18-20°C) throughout development to maintain repression and are subsequently shifted to restrictive temperatures (29-30°C) after eclosion to restore GAL4 activity. At elevated temperatures, GAL80 ^TS^ undergoes a conformational change that destabilises the protein, abolishing its ability to inhibit GAL4 and thereby permitting transgene expression specifically in adults. However, during experiments employing *tubP* -GAL80 ^TS^ expression, we consistently observed that at week 5 post-eclosion, at 29°C, flies consistently exhibited pronounced flight defects that upon further investigation were found to be absent in driver-only controls ( **Fig. 1A** ). Although climbing performance was not significantly affected at this stage, both experimental and control genotypes showed a modest decline in climbing success relative to week 4 post-eclosion ( **Fig. 1B** ). We found that GAL80 ^TS^ had no negative effects on the survival of flies.

Turning to flies with *tubP* -GAL4-driven expression of an RNAi transgene targeting EGFP, we unexpectedly found that flies with this genotype experienced reduced survival as early as week 1 post-eclosion when incubated at the standard temperature of 25°C ( **Fig. 1C** ). Survival defects worsened by week 2 post-eclosion. Similar effects were present when utilising transgenes inserted at site-specific genomic integration sites on either chromosome 2 ( *attP40* ) or chromosome 3 ( *attP2* ), with the latter showing a stronger phenotype ( **Fig. 1C** ). Although we noticed a trend for a greater percentage of non-fliers at week 2 post-eclosion, the difference did not reach statistical significance ( **Fig. 1D** ). However, flies with constitutive expression of *EGFP-RNAi* exhibited marked climbing defects, with the time for the first fly in a population taking significantly longer to reach a pre-determined threshold compared to controls ( **Fig. 1E** ). This effect was observed as early as week 1 post-eclosion and was more pronounced for flies having the chromosome 3 inserted transgene. We found similar phenotypes in flies expressing an RNAi targeting mCherry ubiquitously via the *tubP* -GAL4 driver. Thus, compared to control, flies had significant flight and climbing defects on week 1 post-eclosion, which persisted and even worsened with an increase in age ( **Fig. 1F, G** ). These findings indicate that regulatory and control constructs often presumed to be physiologically inert can, in fact, exert measurable effects on motor behaviour. Consequently, researchers should exercise caution when employing such tools. In particular, behavioural data should be interpreted carefully. Wherever possible, the use of tissue-specific rather than constitutive GAL4 drivers, the avoidance of sensitive timepoints, and the inclusion of appropriate genetic controls are recommended to mitigate confounding effects.

To our knowledge, this is the first report identifying commonly used genetic tools such as GAL80 ^TS^ and RNAi constructs targeting exogenous sequences as having potential toxic effects in *Drosophila* . Although the underlying molecular mechanisms were not directly examined, several hypotheses may explain the observed motor impairments. Overexpression of non-native proteins such as GAL80 ^TS^ could result in protein misfolding or overaccumulation, thereby inducing proteostatic stress. Similarly, while EGFP and mCherry are exogenous sequences, their corresponding RNAi constructs may contain regions of partial complementarity to endogenous transcripts, giving rise to off-target silencing effects. Alternatively, or additionally, elevated levels of double-stranded RNA could provoke cellular stress through activation of innate immune pathways (Chen & Hur, 2022).

It is noteworthy that analogous phenotypes have been reported with other widely used genetic tools. For example, ubiquitous or neuronal expression of GFP has been shown to reduce lifespan and impair locomotor performance in flies (Mawhinney & Staveley, 2011). Furthermore, high levels of GAL4 were found to alter the expression of many *Drosophila* genes in a *UAS* -independent manner (Liu & Lehmann, 2008). More recently, strong expression of GAL4 driven by the yolk-GAL4 promoter in the fat body was reported to cause marked physiological disruptions, including loss of adipose tissue integrity, depletion of lipid stores, reduced fecundity, and increased susceptibility to bacterial infection (Keith et al., 2025).

Collectively, these findings, together with our own, underscore that transgenes and regulatory elements often assumed to be physiologically neutral can exert significant biological effects. They highlight the necessity of rigorous characterisation of genetic tools to ensure accurate experimental interpretation. We recommend that researchers interpret behavioural and physiological phenotypes with caution, incorporate appropriate genetic controls, and, where possible, employ tissue-specific rather than constitutive drivers to minimise unintended consequences. Such precautions are essential for fully harnessing the power of genetically tractable model organisms like *Drosophila* while maintaining experimental rigour and reproducibility.

## Methods

***Drosophila* husbandry and stocks**

Flies were reared at 25°C or, where indicated, at 29°C, on a 12-hour light/dark cycle using a standard sugar-cornmeal-yeast-agar medium. Stocks utilised in this work are listed in the Reagents section and were all obtained from the Bloomington *Drosophila* Stock Center (BDSC). All experimental genotypes were generated using standard genetic crossing schemes.

**Adult fly survival**

Adult flies were maintained in vials at a density of 15–30 flies per vial. The percentage number of flies alive at each time point measured was determined by dividing the number of flies still alive by the initial number of flies in the vial and multiplying the value by 100.

**Flight assay**

Flight performance was assessed using the Droso-Drome apparatus, as previously described (Borg et al., 2023; Herrera & Cauchi, 2023). The device consists of a 1 L glass cylinder coated internally with a sticky, alcohol-based solution and divided into four 5 cm vertical sectors over a total height of 20 cm. Prior to testing, flies were conditioned by inducing at least three consecutive negative geotaxis responses in an empty vial. Conditioned flies were then released from the top of the apparatus, and their landing positions were recorded. The percentage of flies adhering to each sector was calculated, with a distribution skewed toward the lower sectors indicating impaired flight ability. Each group was tested in four independent trials, and a minimum of three groups per genotype were assayed.

**Climbing assays**

Climbing performance was assessed using a negative geotaxis assay. Groups of 15–20 adult flies were transferred into a confined climbing arena constructed from two empty, vertically oriented polystyrene tubes joined end-to-end with tape and allowed to acclimate for 1–2 minutes. Flies were then gently tapped to the bottom of the apparatus to elicit a negative geotaxis response, and the number of flies crossing an 8 cm mark within 10 seconds was recorded. For genotypes with reduced survival, an adapted assay was used. In this version, the time taken for the first fly in a group to cross the 8 cm mark following gentle tapping to the bottom of an empty plastic vial was measured. The assay was terminated if no fly reached the threshold within 120 seconds. In both assays, each group underwent four independent trials, and at least three biological replicates (groups) were tested per genotype.

**Statistical analysis**

All quantitative data are presented as mean ± standard error of the mean (SEM). Statistical analyses were performed using GraphPad Prism version 10.6.0 (GraphPad Software, USA). Two-way ANOVA was used to compare multiple groups followed by Bonferroni’s *post hoc* test. Statistical comparisons for the flight assay were restricted to Sector 1, as this sector most reliably differentiates fliers from non-fliers. Statistical significance was defined as *p* < 0.05.

## Reagents

**Table 1: Table of *Drosophila* stocks used in this study with corresponding identifiers.**

**Table:** 

**Stock**	**Source**	**Stock ID**
* w ^1118^*	BDSC	RRID:BDSC_5905
* tubP-GAL80 ^TS^ ; tubP-GAL4 *	BDSC	RRID:BDSC_86328
*tubP-GAL4*	BDSC	RRID:BDSC_5138
*mCherry-RNAi*	BDSC	RRID:BDSC_35785
*EGFP-RNAi chr 2*	BDSC	RRID:BDSC_41550
*EGFP-RNAi chr 3*	BDSC	RRID:BDSC_41551

**Table 1: Table of software used in this study.**

**Table:** 

**Resource**	**Source**	**Identifier**
Microsoft Excel for Mac version 16.102.3	Microsoft	https://www.microsoft.com/en-us/microsoft-365/excel
GraphPad Prism version 10.6.1	GraphPad Software	https://www.graphpad.com
